# Novel sheath-assisted endoscopic ultrasound-guided drainage for pericholecystic abscess

**DOI:** 10.1055/a-2072-3830

**Published:** 2023-04-26

**Authors:** Koichiro Mandai, Yukari Kanemitsu, Shiho Nakamura

**Affiliations:** Department of Gastroenterology, Kyoto Second Red Cross Hospital, Kyoto, Japan

Recently, a newly designed endoscopic sheath (outer diameter, 8.5 Fr; inner diameter, 6 Fr; UMIDAS sheath cannula; UMIDAS Inc, Kanagawa, Japan) has become available in Japan ( Fig. 1 ). Here, we describe a novel sheath-assisted endoscopic ultrasound (EUS)-guided drainage procedure for a pericholecystic abscess.

**Fig. 1:**
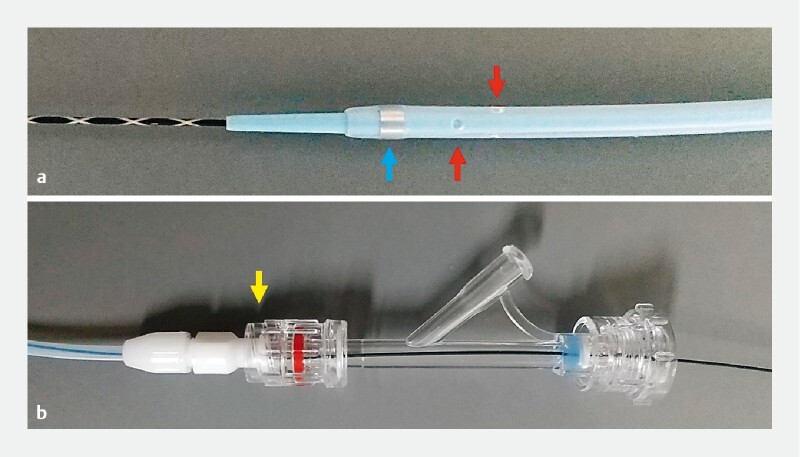
Images of a novel endoscopic sheath (UMIDAS sheath cannula). **a** An 8.5-Fr outer sheath has a radiopaque marker (blue arrow) and side holes (red arrow) near the tip. **b** A Y-connector, attached to the proximal end of the outer sheath (yellow arrow), allows contrast medium injection under guidewire loading.

A 90-year-old man was referred to our hospital because of multiple pericholecystic abscesses caused by acute cholecystitis ( Fig. 2 ). EUS-guided abscess drainage was performed; however, he had a persistent high fever due to residual abscess. Therefore, EUS-guided drainage was attempted. Using an echoendoscope, we visualized the abscess from the antrum of the stomach. Gel immersion was used to prevent double mucosal puncture 1 , and the fluid space of the abscess – clearly visualized under contrast-enhanced harmonic EUS – was punctured using a 19-gauge needle ( Fig. 3 ). After aspirating yellowish white pus, a 0.025-inch guidewire was inserted in the abscess. After removing the needle, the fluid drained in the stomach through the needle track without leaking into the abdominal cavity, indicating adhesion between the gastric wall and abscess. The needle track was dilated using a 7-Fr spiral dilator (Tornus ES; Asahi Intecc Co., Aichi, Japan). Next, the novel endoscopic sheath (UMIDAS sheath cannula) was inserted within the abscess for irrigation and subsequent stent insertion. The inner catheter was removed, and the outer sheath and guidewire were left in place ( Fig. 4 a ). Intra-abscess irrigation with sterile saline was performed through the side port of the outer sheath. Subsequently, a 30-cm long plastic stent, made by cutting a 5-Fr endoscopic nasobiliary drainage tube 2 , was inserted through the outer sheath and placed from the abscess to the stomach ( Fig. 4 b , Video 1 ).

**Fig. 2:**
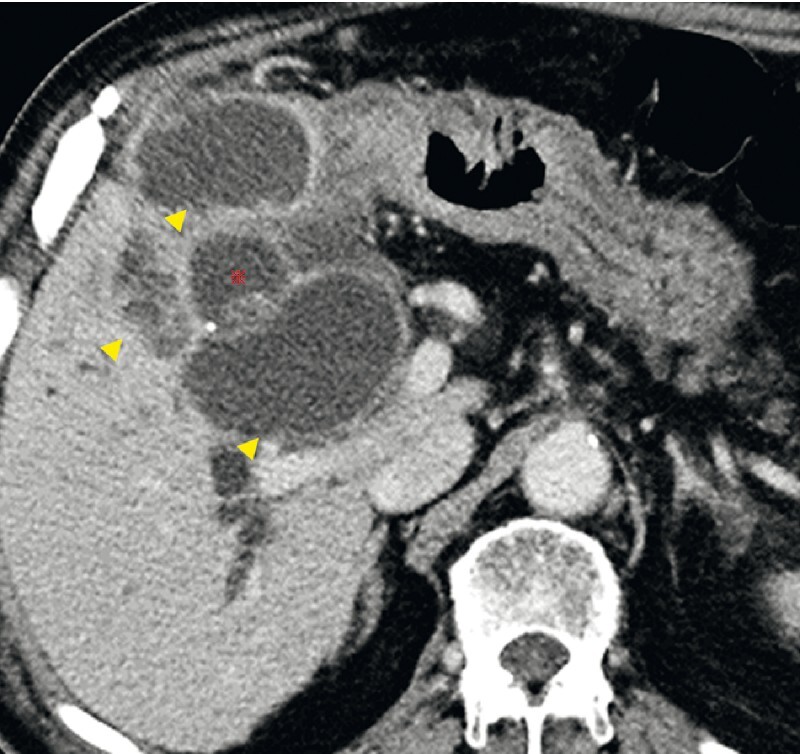
Computed tomography shows the multiple abscess (yellow arrowheads) around the gallbladder (red asterisk).

**Fig. 3:**
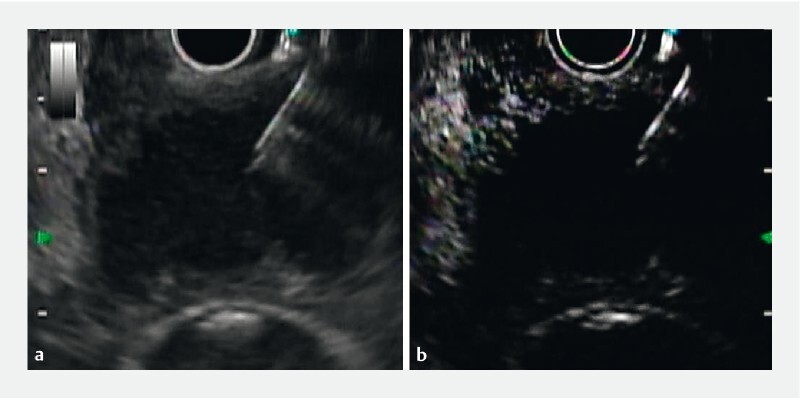
Endoscopic ultrasonography shows the fluid space of the abscess, which was punctured using a 19-gauge needle. **a** B-mode view. **b** Contrast-enhanced view.

**Fig. 4:**
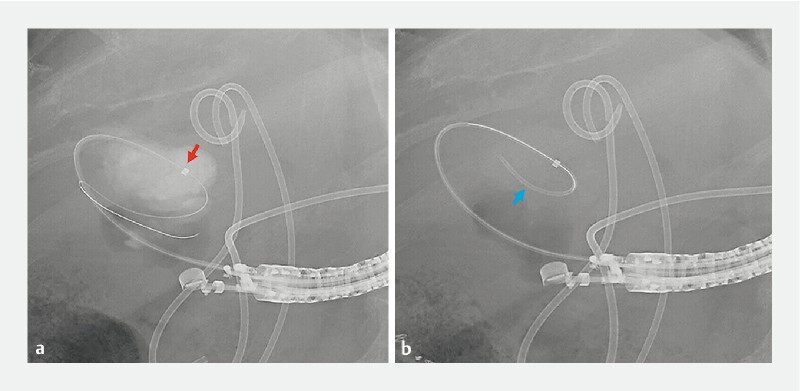
Fluoroscopic images. **a** The outer sheath (red arrow) and the guidewire were left in the abscess. **b** A 5-Fr plastic stent (blue arrow) was inserted through the outer sheath.

**Video 1** Endoscopic ultrasound-guided drainage for pericholecystic abscess. Intra-abscess irrigation and 5-Fr plastic stent placement through the novel endoscopic sheath.


This technique may be useful for EUS-guided abscess drainage. However, in cases without adhesion to the stomach, it should be performed with a covered metal stent to prevent fluid leakage into the abdominal cavity.

Endoscopy_UCTN_Code_TTT_1AS_2AD

## Notes

Endoscopy E-Videos

https://eref.thieme.de/e-videos
*E-Videos* is an open access online section of the journal *Endoscopy* , reporting on interesting cases and new techniques in gastroenterological endoscopy. All papers include a high-quality video and are published with a Creative Commons CC-BY license. Endoscopy E-Videos qualify for HINARI discounts and waivers and eligibility is automatically checked during the submission process. We grant 100% waivers to articles whose corresponding authors are based in Group A countries and 50% waivers to those who are based in Group B countries as classified by Research4Life (see: https://www.research4life.org/access/eligibility/ ). This section has its own submission website at https://mc.manuscriptcentral.com/e-videos

## References

[1] Mandai K Yoshimoto T Uno K Gel immersion‐assisted endoscopic ultrasound‐guided gallbladder drainage using a fine‐gauge electrocautery dilator Dig Endosc 2022 34 e155 e156 35983719 10.1111/den.14411

[2] Mandai K Shinomiya R Uno K Transpapillary biliary drainage using a long plastic stent: Preventing early stent dysfunction in pancreatic cancer with duodenal invasion J Hepatobiliary Pancreat Sci 2022 29 e52 e53 35114068 10.1002/jhbp.1121

